# Supplementary material for: Inferring Adaptive Introgression Using Hidden Markov Models
Source: Mol Biol Evol. 2021 Jan 27;38(5):2152–65. doi: 10.1093/molbev/msab014 (PMC8097282; doi:10.1093/molbev/msab014)
Supplement: msab014_Supplementary_Data [file msab014_supplementary_data.pdf]

Inferring Adaptive Introgression Using Hidden Markov Models

Svedberg et al.

Supplementary materials

Supplementary figures

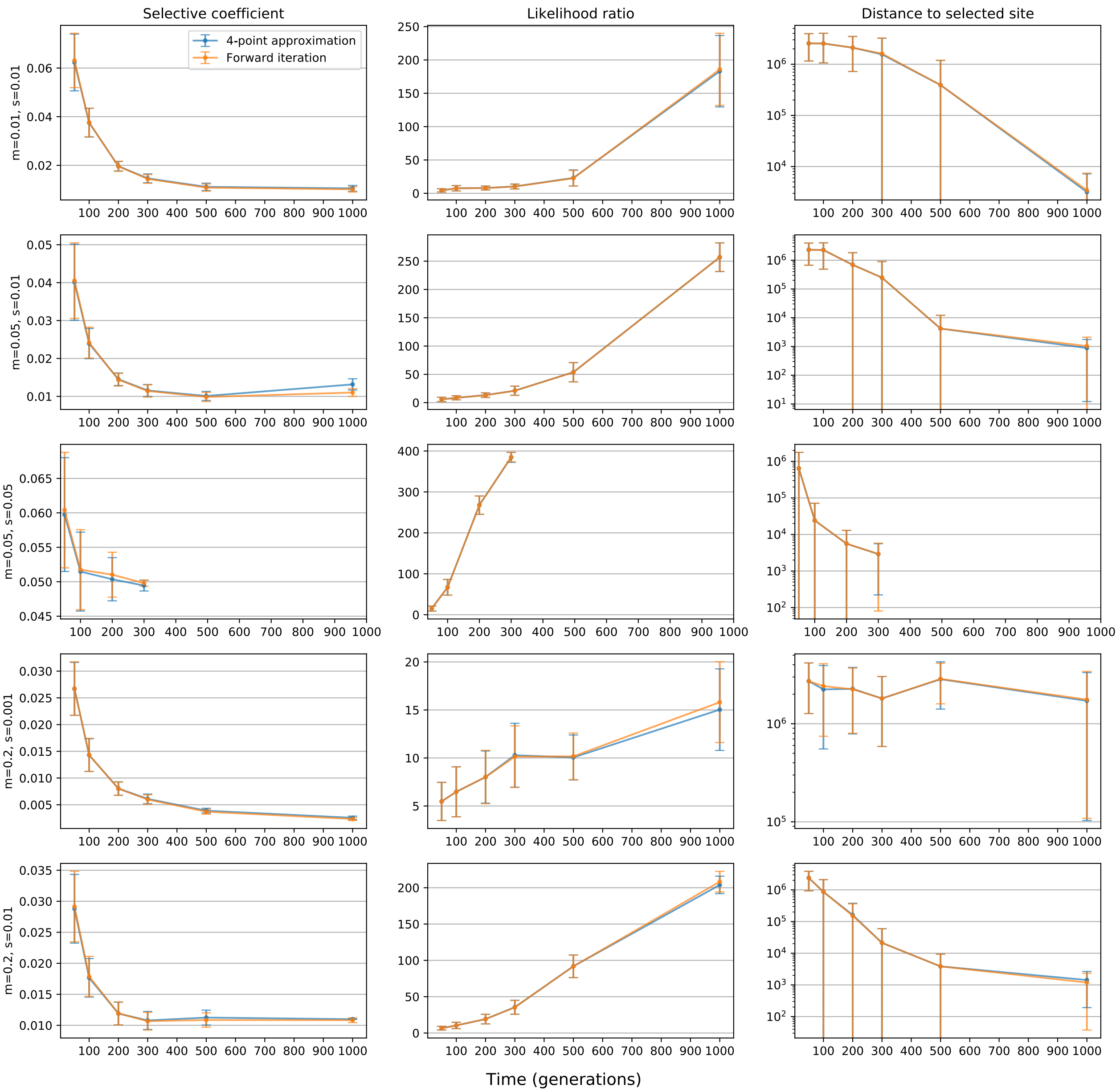

Figure S1: Comparison of the two different algorithms implemented in AHMM-S for calculating Markovian transition rates surrounding a selected locus
The forward iteration method will iterate through each site on a chromosome and calculate the expected transition rates between them assuming a logistic allele frequency trajectory for the selected site. In the faster 4-point analytical approximation, only the transition rates between 4 points on each side of selected sites are calculated, and the transition rates of the rest of the sites can then be interpolated. These two methods perform in close to identical fashion over all parameters that have been compared and we therefore use the 4-point approximation as our default to facilitate rapid computation.

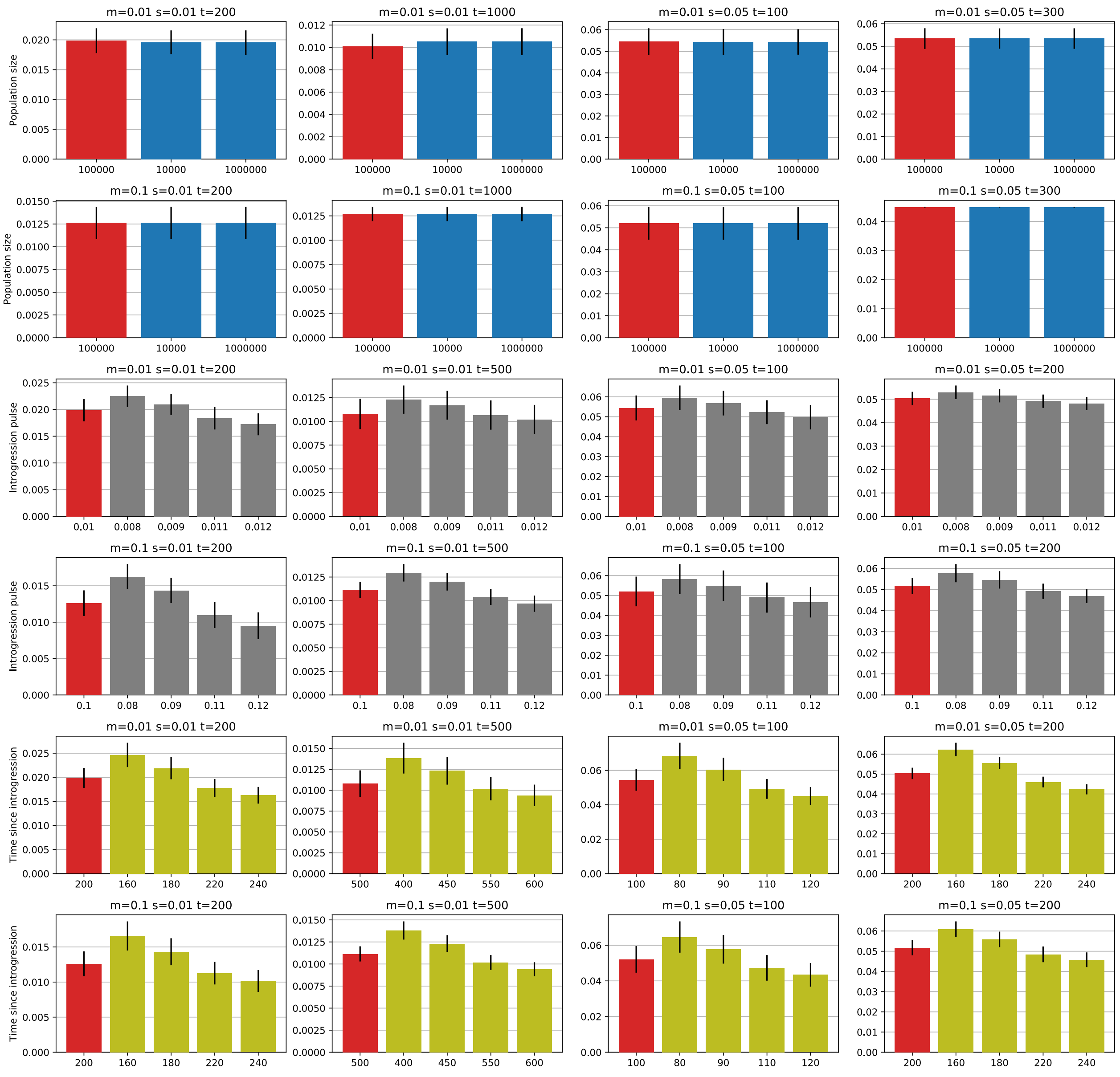

Figure S2: Effects of misspecifying the size of the population, the size of the introgression pulse and the time since introgression in generations on the estimated selective coefficient

The true value is shown in red. The model is robust for misspecification of population size (blue bars), where using a value an order of magnitude larger or smaller has insignificant effects. For the size (grey bars) and timing of introgression (green bars), the effects are larger, with misspecifications of ~20% generating errors of 20-40%. Overestimating these parameters leads to an underestimated value of s, and vice versa.

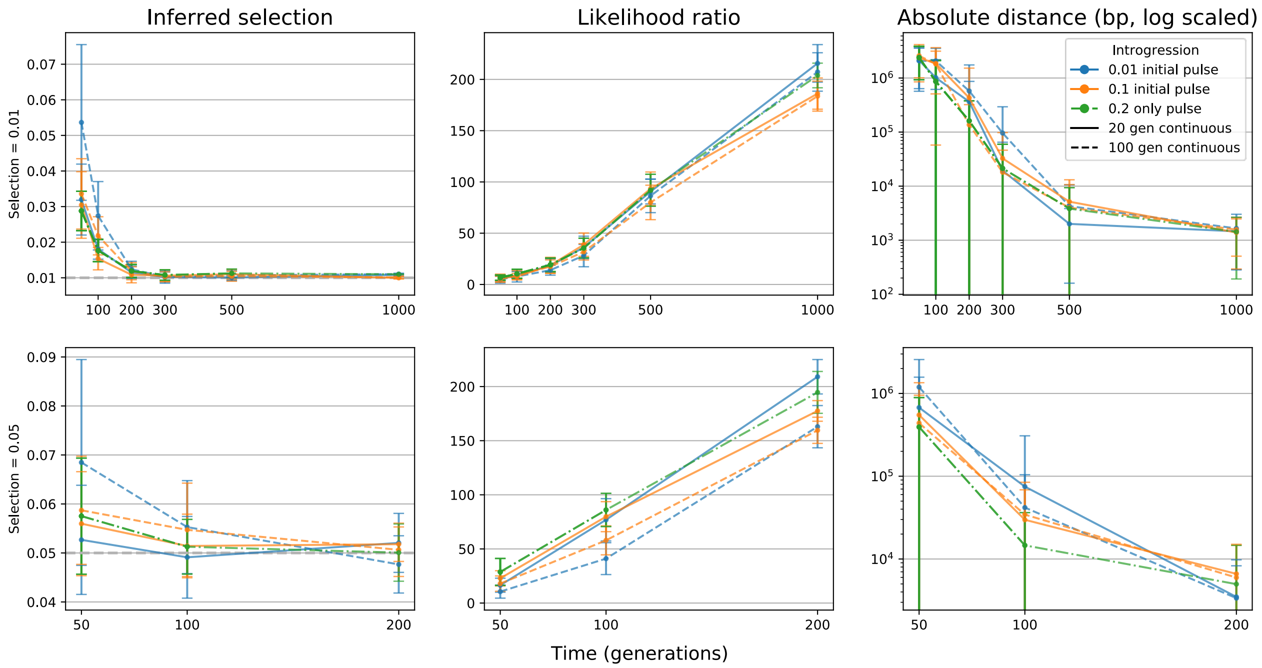

Figure S3: Continuous gene flow

We performed simulations where after an initial introgression pulse of 1% or 10%, gene flow continued at 1% per generation for 20 generations, or 0.2% per generation for 100 generations. The selected allele had s=0.01 or s=0.05. The final background introgression fraction was in all cases ~20%, and we compare these simulations to a single 20% pulse. The x axis shows time in generation since introgression and the y axis either the inferred value of s, the likelihood ratio or the distance between the inferred position and the real position.

Continuous gene flow had little effect on both the ability to identify the correct locus and on estimating the selective coefficient. At early time points, especially when gene flow was still ongoing, estimates of *s* was higher than with a single introgressive pulse, but from 100-200 generations, the differences were minor.

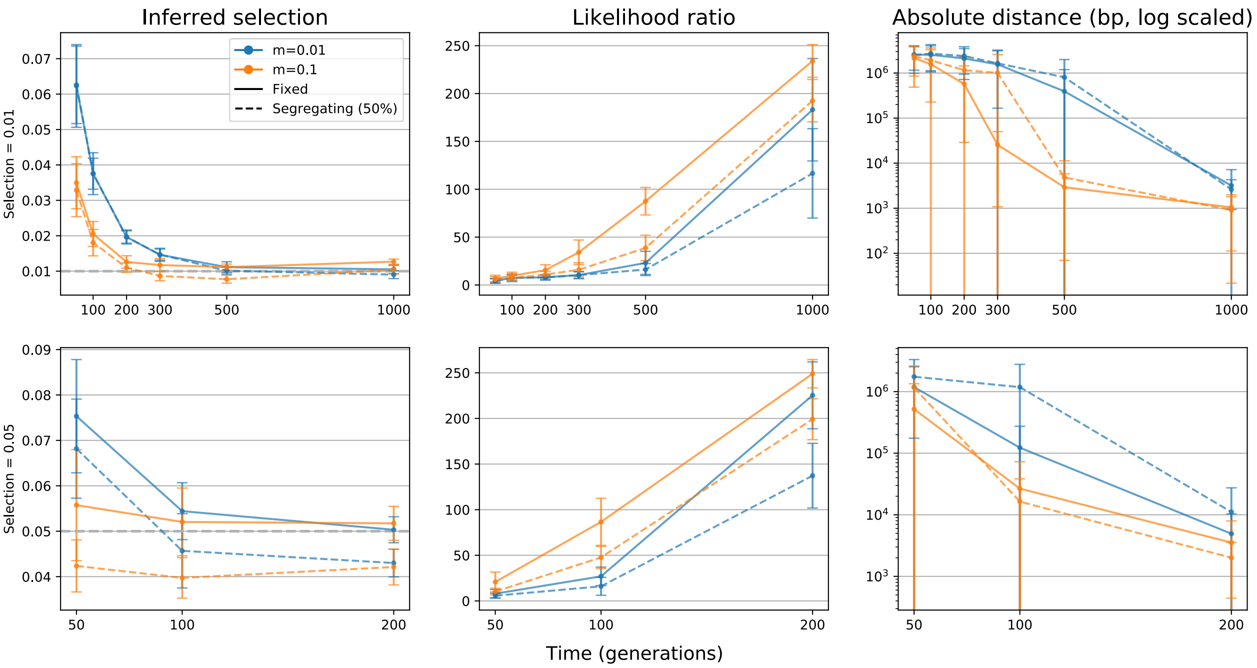

Figure S4: Selected site segregating in donor population

We performed simulations of an adaptive introgression scenario where the adaptive allele is segregating at 50% in the donor population. The introgression pulse in the simulations were either 1% or 10%, and s was either 0.01 or 0.05. The x axis shows time in generation since introgression and the y axis either the inferred value of s, the likelihood ratio or the distance between the inferred position and the real position.

When the selected site is segregating in the donor population, AHMM-S infers the position with less accuracy at intermediate time points, but as the time since admixture increases the difference in precision compared to when the site is fixed diminishes. The selective coefficient is generally underestimated, with values up to 20% below the true value.

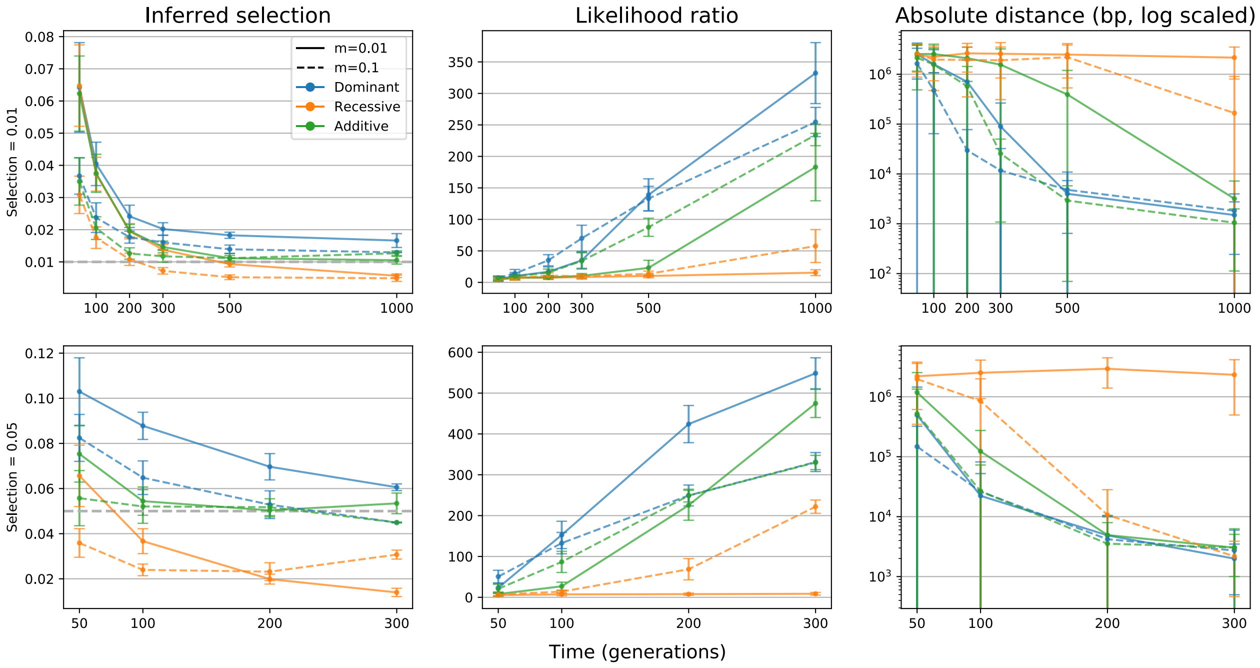

Figure S5: Dominant vs recessive vs additive selection

Results for simulations where the selected allele acted as a dominant, recessive or additive locus. (All other simulations use additive selection). Introgression pulse in the simulations was either set to 1% or 10% and the selection coefficient s to 0.01 or 0.05. The x axis shows time in generation since introgression and the y axis either the inferred value of s, the likelihood ratio or the distance between the inferred position and the real position.

Dominant selection appears to improve the precision in identifying the correct position (probably due to increasing in frequency more quickly), but the estimated value of *s* is generally higher than the true value. An allele that acts recessively will conversely show the opposite pattern, most likely because it increases in frequency more slowly. Especially in the case where the introgressive pulse is only 1%, we are never able to identify the site with adequate precision, probably due to the introgressed allele only very rarely being found in a homozygous state.

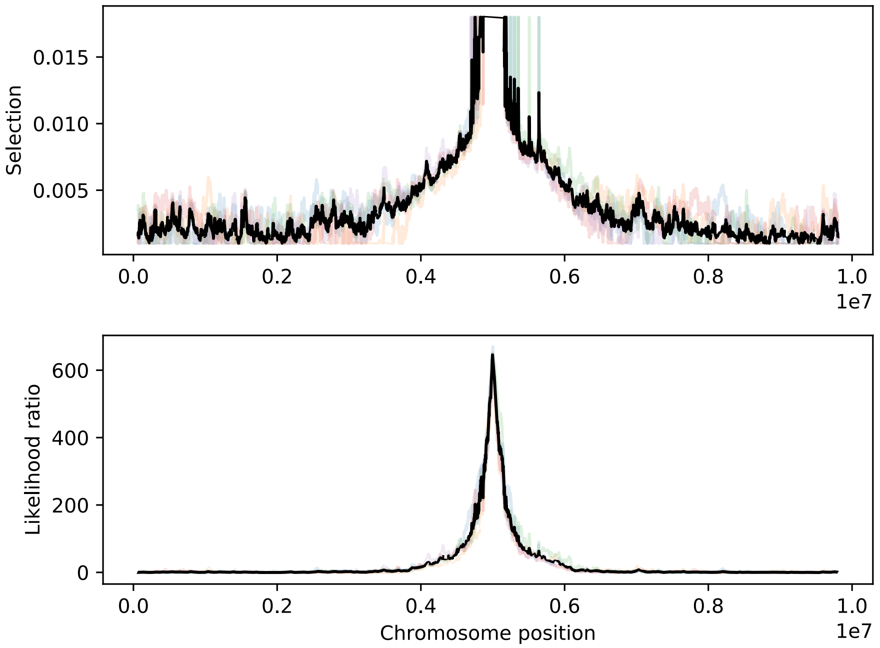

Figure S6: Example of AHMM-S analysing an adaptive introgression scenario where the selected site has gone to fixation

Inferred selected coefficient and likelihood ratio from five simulations (and the mean of the five simulations) of a 1% introgression of an allele with s=0.05, sampled after 1000 generations. The likelihood ratio peak identifies the correct position with high accuracy, but AHMM-S will only report a peak selection coefficient of ~0.017, as that is the value of s that causes a site to go to fixation at 1000 generations.

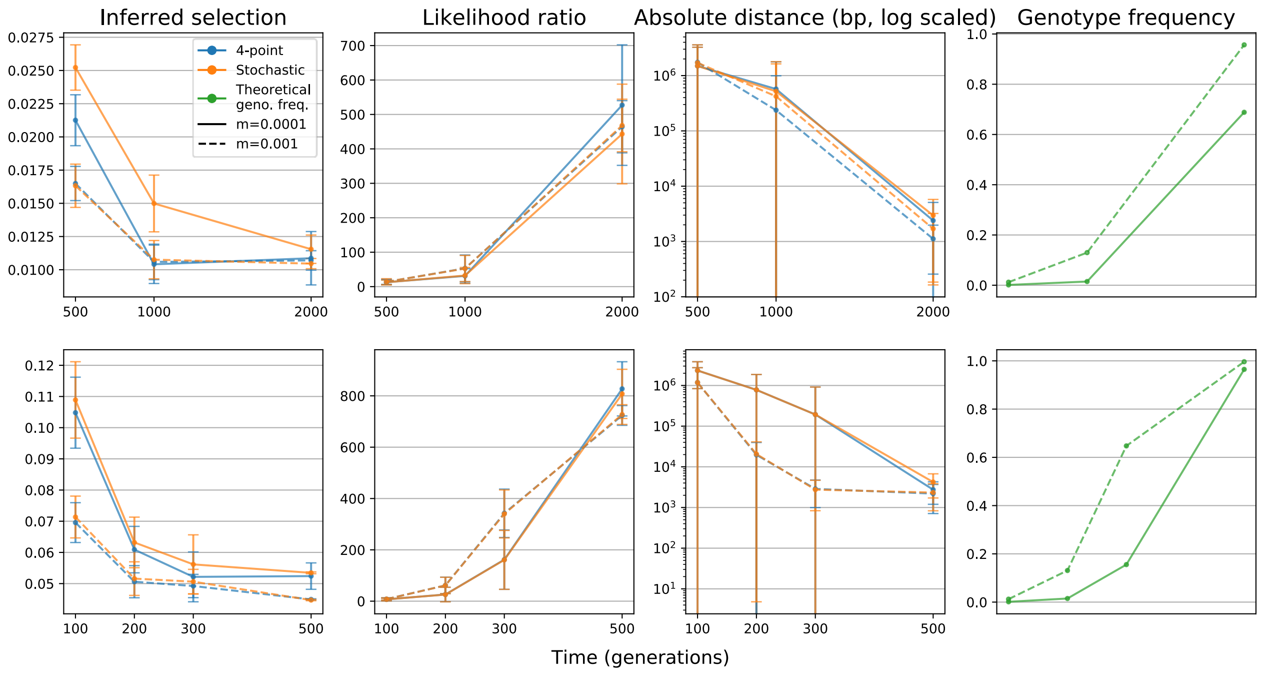

Figure S7: Small introgression fractions

Results for simulations with a low introgression fraction. We compared the performance of the 4-point approximative algorithm for calculating the expected increase in frequency of an adaptively introgressed allele, with a stochastic algorithm based on averaging multiple forward simulations. Small introgression fractions is expected to violate the model used for the 4-point approximation, but it performs equally well as the more precise (but significantly slower) stochastic method.

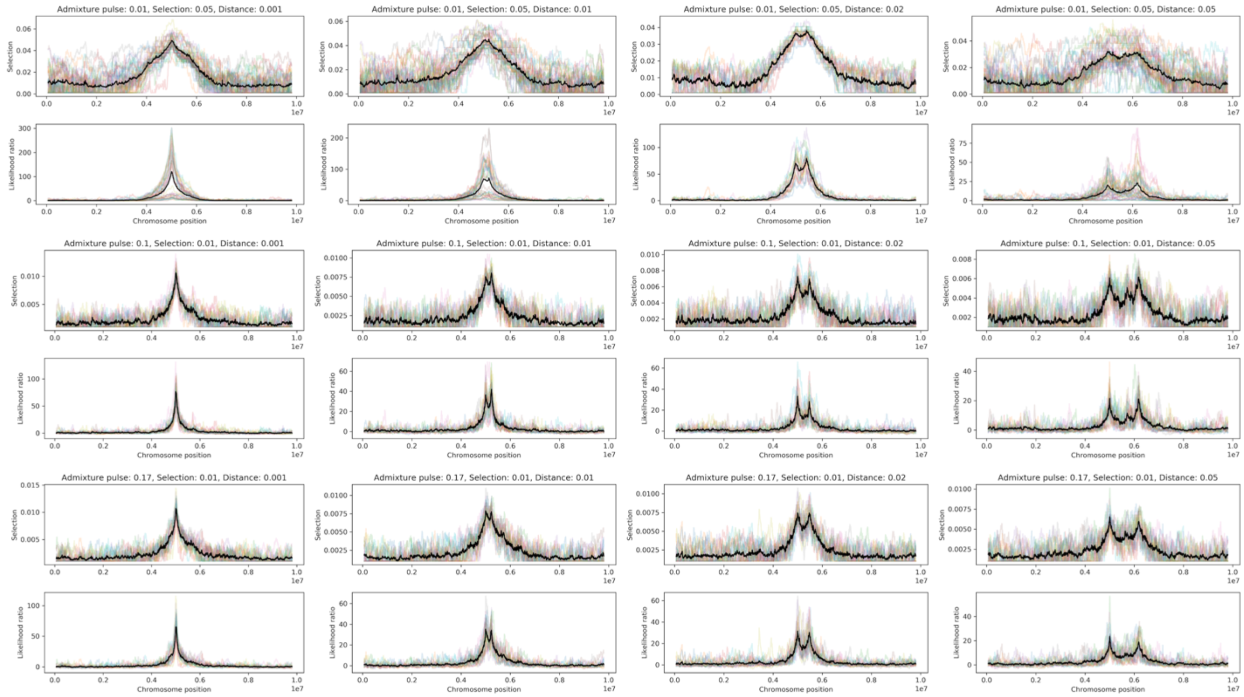

Figure S8: Peak separation for closely linked selected sites

Three different introgression scenarios were simulated (A: m=0.1, s=0.01 t=500; B: m=0.01, s=0.05, t=200; C: m=0.17, s=0.01, t=430) in 20 replicates with two sites of equal selective strength (s/2) located at increasing distances (0.1, 1, 2 and 5 cM) from each other. Two distinct peaks are visible in the cases of 1, 2 and 5 cM for all three scenarios.

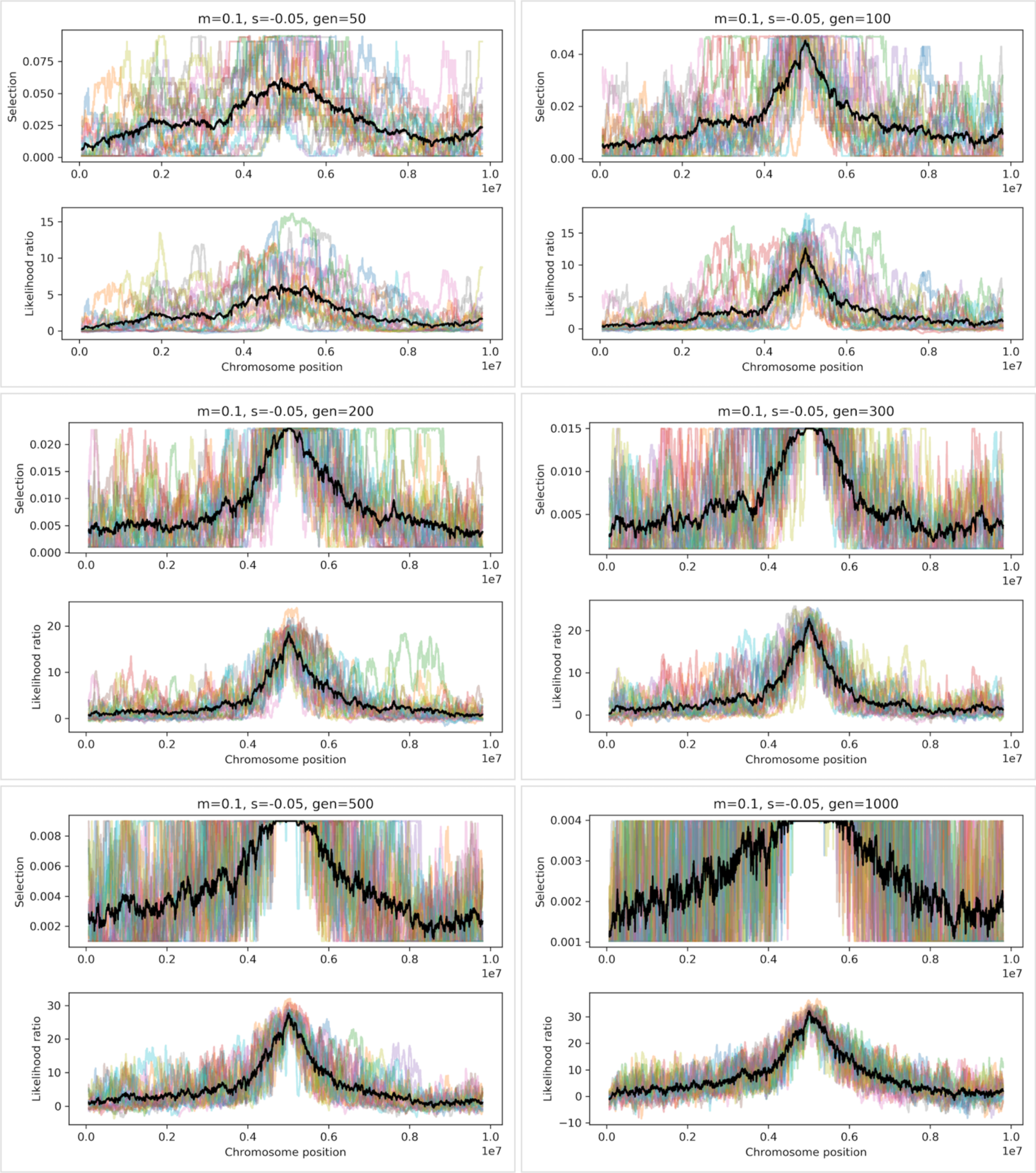

Figure S7: Negative selection

Example of a simulation of an introgressed site experiencing negative selection, with an introgressive fraction of 0.1 and a selective coefficient of 0.05. We ran 20 simulations and sampled them from 50 to 1000 generations. We analyzed the simulations with AHMM-S after reverting the genotype identity, so the negative selection of the introgressed site shows up as positive selection of the receiving genotype. AHMM-S can identify the location of the selected site, but as the frequency of the site over time approaches zero, the precision of the estimated selective coefficient is lost.

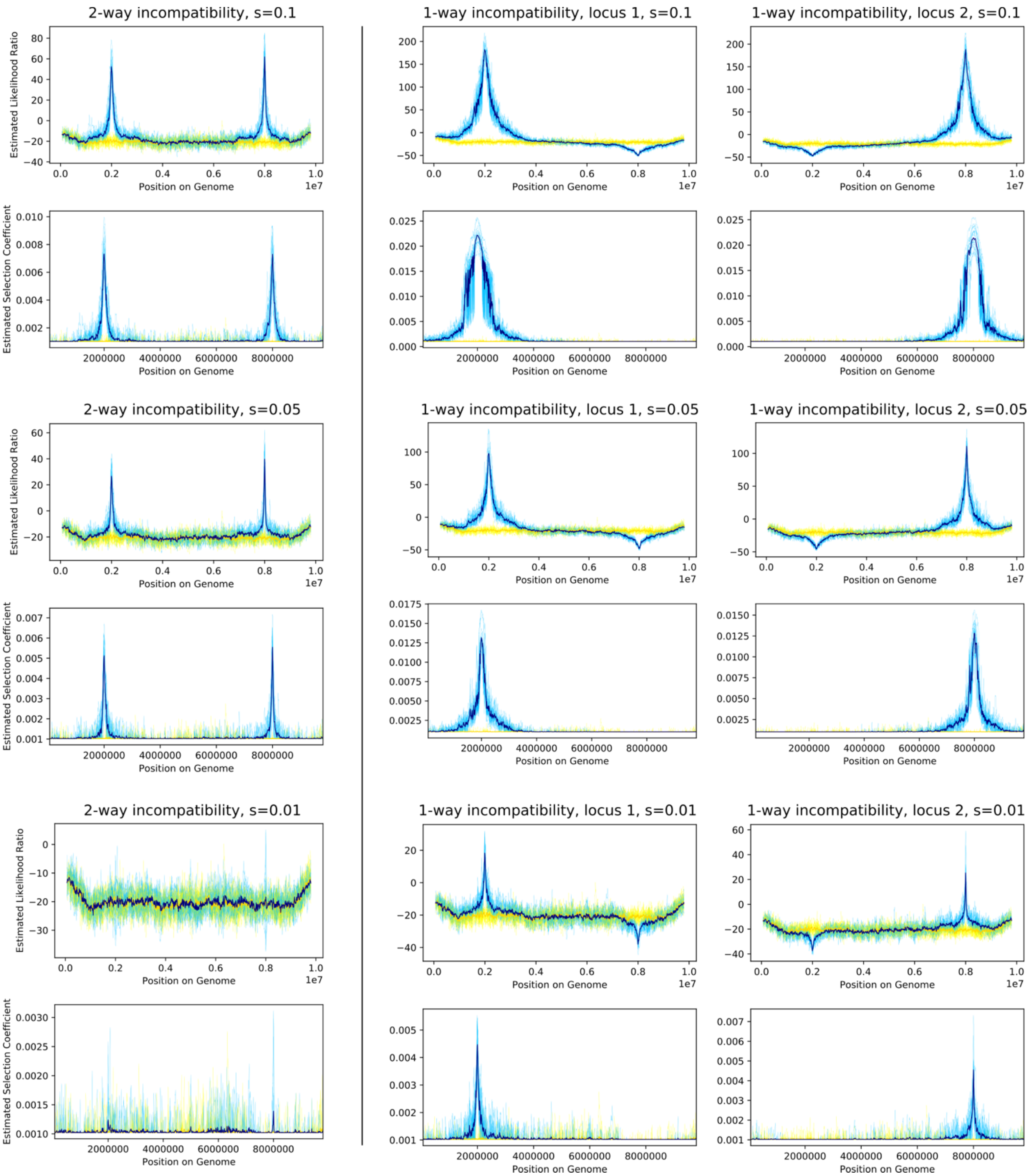

Figure S10: Dobzhansky-Muller Incompatibilities (DMIs) identified using AHMM-S
Two scenarios were investigated for three different values of s (-0.1, -0.05 and -0.01) with an introgressive pulse of 0.5. In both scenarios, two loci (A and B), located at 2 Mbp and 8 Mbp, have an incompatibility interaction between alleles from the two different populations. In scenario 1 (column 1), any interaction between different alleles is selected against (A0B1 and A1B0), in scenario 2 (column 2 and 3) only one combination (A0B1) is selected against. The

interacting loci could be identified in all cases except scenario 1, s=0.01. The results for scenario 2 are split into two columns as the direction of selection is different for each locus and AHMM-S must identify each locus separately in different analyses. Blue lines show simulations with DMI and yellow lines negative controls without any active selection. Darker lines show mean values and lighter lines the results for each simulation. Negative likelihood ratios can appear when the likeliest inferred value of s is less likely than the neutral case. This can be explained by the search space for s starting at 0.001.

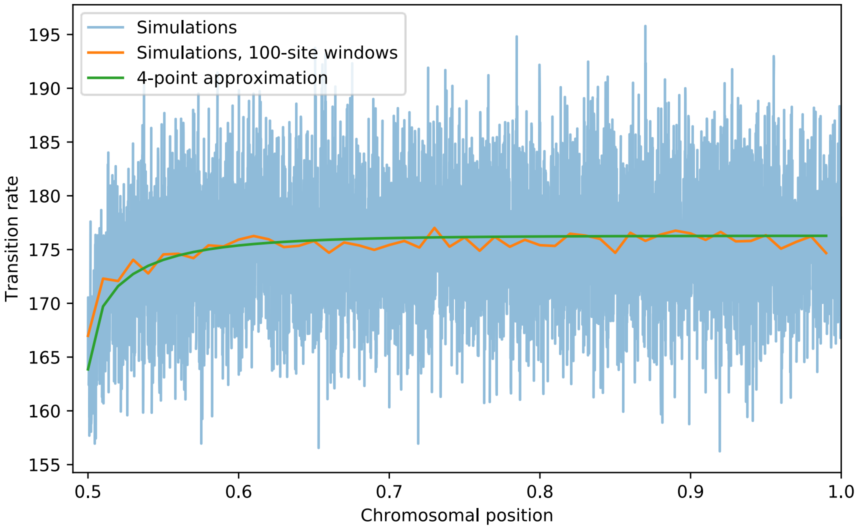

Figure S11: Simulated vs expected transition rates

Transition rates for 100,000 simulations are plotted at each site (blue), or averaged in 100-site windows (orange), and compared to transition rates estimated with the 4-point approximation method (green). The selected site is located at position 0.5 and the transition rates are plotted going away from the selected site to the end of the chromosome at position 1.

Supplementary tables

Table S1: GO categories including more than 10 candidate genes

GO Term	Number of genes	Q-value (FDR corrected)	Description	Genes Detected
GO:0097159	21	0.094285	organic cyclic compound binding	tRNA:Arg-ACG-1-4, osa, Cyp6a22, Cyp6w1, tRNA:Lys-CTT-1-5, RIOK2, tRNA:Arg-ACG-1-3, tRNA:Arg-ACG-1-5, Cyp6a17, trx, Cyp6g1, SmF, scaRNA:MeU2-C41, dsx, Pcf11, EndoG, GlnRS, CG10445, CG2678, lds, cic
GO:1901363	21	0.094285	heterocyclic compound binding	tRNA:Arg-ACG-1-4, osa, Cyp6a22, Cyp6w1, tRNA:Lys-CTT-1-5, RIOK2, tRNA:Arg-ACG-1-3, tRNA:Arg-ACG-1-5, Cyp6a17, trx, Cyp6g1, SmF, scaRNA:MeU2-C41, and dsx, Pcf11, EndoG, GlnRS, CG10445, CG2678, lds, cic
GO:0003676	13	0.4480193103	nucleic acid binding	tRNA:Arg-ACG-1-4, osa, tRNA:Lys-CTT-1-5, tRNA:Arg-ACG-1-3, tRNA:Arg-ACG-1-5, trx, SmF, scaRNA:MeU2-C41, dsx, Pcf11, EndoG, CG2678, cic
